# Dual-Mode Gas Sensor Composed of a Silicon Nanoribbon Field Effect Transistor and a Bulk Acoustic Wave Resonator: A Case Study in Freons

**DOI:** 10.3390/s18020343

**Published:** 2018-01-25

**Authors:** Ye Chang, Zhipeng Hui, Xiayu Wang, Hemi Qu, Wei Pang, Xuexin Duan

**Affiliations:** 1 State Key Laboratory of Precision Measuring Technology & Instruments, Tianjin University, Tianjin 300072, China; cy0803@tju.edu.cn (Y.C.); xy_wang@tju.edu.cn (X.W.); weipang@tju.edu.cn (W.P.); 2 China Marine Development and Research Center (CMDRC), Beijing 100049, China; hzpeng117@163.com

**Keywords:** freons detection, bulk acoustic wave resonator, field effect transistor, dual-mode sensing, gas sensor

## Abstract

In this paper, we develop a novel dual-mode gas sensor system which comprises a silicon nanoribbon field effect transistor (Si-NR FET) and a film bulk acoustic resonator (FBAR). We investigate their sensing characteristics using polar and nonpolar organic compounds, and demonstrate that polarity has a significant effect on the response of the Si-NR FET sensor, and only a minor effect on the FBAR sensor. In this dual-mode system, qualitative discrimination can be achieved by analyzing polarity with the Si-NR FET and quantitative concentration information can be obtained using a polymer-coated FBAR with a detection limit at the ppm level. The complementary performance of the sensing elements provides higher analytical efficiency. Additionally, a dual mixture of two types of freons (CFC-113 and HCFC-141b) is further analyzed with the dual-mode gas sensor. Owing to the small size and complementary metal-oxide semiconductor (CMOS)-compatibility of the system, the dual-mode gas sensor shows potential as a portable integrated sensing system for the analysis of gas mixtures in the future.

## 1. Introduction

Micro sensors are small-sized devices with flexibility, high sensitivity, moderate cost, and reduced power consumption. During the past decades, a variety of micro sensors have been employed in gas-sensing applications [1,2], including gravimetric [3], electrochemical [4,5], and optical [6] sensors. Recent studies in systems based on micro gas sensors have demonstrated their capabilities in analyzing breath samples for disease diagnosis [7,8], assessing the authenticity of premium products [9], evaluating the freshness and maturity of food [10,11], assuring quality in product manufacturing [12], etc. However, in most applications, the sensing system is composed of one type of sensor or utilizes one type of transduction mode, which only discloses partial information about the analytes. The combination of different types of sensors or transduction modes could provide complementary information to improve the sensor’s selectivity and facilitate the discrimination of gas mixtures [13,14,15].

A field effect transistor (FET) is a type of electrochemical sensor which offers charge-based information, such as the dipole moment inherent in analytes. To date, different types of nanostructure-based FETs, such as nanowires [16,17], nanorods [18,19], nanotubes [20,21], and nanoribbons [22] have been applied for gas sensing. In particular, silicon nanoribbon field effect transistors (Si-NR FETs) fabricated using complementary metal-oxide semiconductor (CMOS)-compatible processes have demonstrated high effectiveness and a superb signal-to-noise ratio, with the limit of detection (LOD) as low as sub-ppm [23]. Thus, the device is reliable in polarity sensing even when the analytes are at the trace level. A bulk acoustic wave resonator (BAW) is a type of gravimetric sensor. The sensitivity and selectivity of a BAW can be tuned by chemical surface coating. The gravimetric-based measurement leads to a sensing signal, which carries information about the adsorption of analytes [24]. In recent years, rapid progress has been made in the field of gas sensing using thin film bulk acoustic resonators (FBARs) [25,26,27,28,29]. Due to their GHz-level resonant frequency and high quality-factor, FBARs show higher sensitivity than conventional acoustic wave devices [30,31].

Freons are halocarbon-based chemicals extensively used as refrigerants, foaming agents, and cleaning solvents for domestic and industrial applications. The excessive emission of freon gases, including partially halogenated hydrochlorofluorocarbons (HCFCs) and chlorofluorocarbons (CFCs), is considered destructive to the ozone layer and contributes to the greenhouse effect [32]. Table 1 lists the physical properties of two typical freons (CFC-113 and HCFC-141b) and their effects on the atmospheric environment. Since those two freons possess similar physical properties, they are difficult to distinguish [33]. Conventional methods of analyzing freons are gas chromatography-mass spectrometry (GC-MS) [34,35] and Fourier transform infrared (FTIR) spectroscopy [36,37]. These methods can provide qualitative and quantitative analysis of freons; however, they are limited by their bulky size and high capital cost for on-line or portable gas analysis [38,39].

In this work, we fabricated *p*-type Si-NR FETs as well as 2.44 GHz FBAR sensors, and incorporated these two types of sensors into a dual-mode gas sensing system for the analysis of CFC-113 and HCFC-141b. Experimental results show that qualitative discrimination can be realized by the Si-NR FET due to its polarity-sensitivity, while the quantitative concentration detection of freons can be realized by a polymer-coated FBAR through gravimetric measurement. Moreover, freon mixtures are analyzed based on the complementary characteristics of the two types of sensors. Our results suggest that the proposed dual-mode gas sensor has potential for the quick and accurate detection of CFC-113 and HCFC-141b, as well as other dual-component gas mixtures, in the future.

## 2. Materials and Methods

### 2.1. Chemicals

Freons (CFC-113 and HCFC-141b), ethanol, hexane, and polyisobutene (PIB, Mw = 600,000) utilized in this work were purchased from J&K Chemical (Beijing, China) in analytical purity (99%) and used without further purification.

### 2.2. Experimental Setup

**Figure 1:**
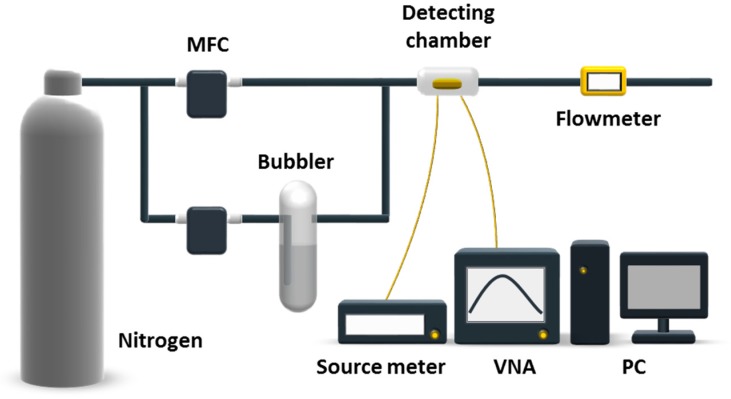
Illustration of the experimental setup for the detection of freons at P/P_0_ from 0.1 to 0.6 with both silicon nanoribbon field effect transistor (Si-NR FET) and thin film bulk acoustic resonator (FBAR) sensors. MFC: mass flow controller; VNA: vector network analyzer; PC: personal computer.

Figure 1 shows the experimental setup used in this work. The setup consists of two parts, a dual-line vapor delivery system and a testing system. In the vapor delivery system, vapors were bubbled out of liquid with carrier nitrogen gas (99.999%). Freon vapor in varied concentrations was prepared by adjusting the flow rate of nitrogen from the dilution line via a mass flow controller (MFC, 5850e, Brooks, Pacific Grove, CA, USA). A flowmeter was used to monitor the real-time flowrate at the end of the system. Experiments were performed with exposure to freon vapors at concentrations in terms of P/P_0_ from 0.1 to 0.6, where P and P_0_ represent the partial pressure and the saturated vapor pressure of the vapor of interest, respectively. According to [42], concentrations of vapors at P/P_0_ from 0.1 to 0.6 are calculated to be at a several thousand ppm level. Freon vapors at lower concentrations ranging from 1 to 20 ppm were produced by a commercial vapor generator system (MF-3D, NIM, Beijing, China). In the testing system, a Si-NR FET was wirebonded onto a 28-pin dual in-line package and source-drain as well as gate voltages (V_g_) were applied using a source meter (2636, Keithley, Taunton, MA, USA). The source-drain current (I_ds_) was measured with the source meter. Meanwhile, a FBAR sensor was wirebonded onto an evaluation board and connected to a vector network analyzer (VNA, E5071C, Agilent, Palo Alto, CA, USA) for frequency domain measurements. The two types of sensors were fabricated independently and packed together in a stainless-steel chamber after fabrication. Real-time sensing data of both sensors were recorded by a personal computer (PC). Ultraviolet light illumination was performed after each experiment to induce freon molecules desorption and keep the sensor surface fresh.

### 2.3. Device Fabrication and Functionalization

**Figure 2:**
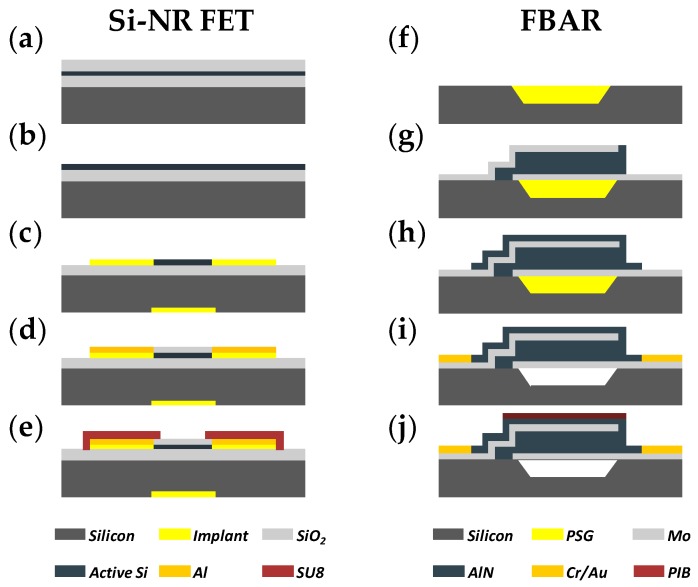
Schematics of the fabrication process of (**a**–**e**) Si-NR FET and (**f**–**j**) FBAR. (**a**) Thermal oxidation of wafer; (**b**) thinning of silicon active layer; (**c**) implantation of source, drain, and back-gate and etching of nanoribbon; (**d**) deposition of front oxide and contact pads; (**e**) deposition of passivation layer; (**f**) etching of air cavity and deposition of passivation layer; (**g**) deposition of sandwiched structure; (**h**) deposition of passivation layer; (**i**) deposition of contact pads and release of phosphosilicate glass (PSG); (**j**) spin-coating of polyisobutene (PIB).

As shown in Figure 2a–e, the *p*-type Si-NR FET in this work was fabricated by top-down lithographic techniques on a 4 inch silicon-on-insulator wafer as described in [43]. Briefly, the silicon active layer (boron-doped, carrier concentration = 10^15^ cm^−3^) was thinned to 45 nm using thermal oxidation and a buffered oxide etch. Subsequently, the source and drain regions, as well as the back-gate, were patterned and further doped by BF_2_^+^ implantation. The nanoribbon mesas (1 × 10 μm^2^ sensing area) were then defined by optical lithography and reactive ion etching (RIE) using Cl_2_ inductively coupled plasma. An oxidation furnace was used to grow a 20 nm thick layer of silicon oxide over the wafer. Metallization was then performed by evaporating Al and patterning by lift-off. After annealing, the metal contacts were measured to ensure ohmic contacts. Finally, a SU-8 layer was patterned to passivate the devices, with openings to NRs and contact pads. 

As shown in Figure 2f–j, the 2.44 GHz FBAR was fabricated with a standard microelectromechanical system (MEMS) fabrication process as described before [26]. First, an air cavity was etched on a single-side polished silicon wafer by deep reactive ion etching (DRIE). Subsequently, the cavity was filled with phosphosilicate glass (PSG) as a sacrificial layer via chemical vapor deposition (CVD). A sandwiched structure of Mo/AlN/Mo was then deposited as the bottom electrode, piezoelectric layer, and top electrode, respectively. After the deposition of a passivation layer (AlN), a Cr/Au composite film was then deposited and patterned to form contact pads. Finally, PSG in the cavity was removed by immersing the chip in a diluted HF solution. After fabrication, the FBAR chip was spin-coated with a layer of PIB (1 mg/mL in chloroform; 4000 rpm for 30 s) and annealed at 65 °C for 1 h. The PIB layer was then characterized by FTIR spectroscopy (Vertex 70v, Bruker Optics, Ettlingen, Germany) and atomic force microscopy (AFM, Dimension Icon, Bruker, Rheinstetten, Germany) in tapping mode.

## 3. Results

### 3.1. Sensor Characterizations

**Figure 3:**
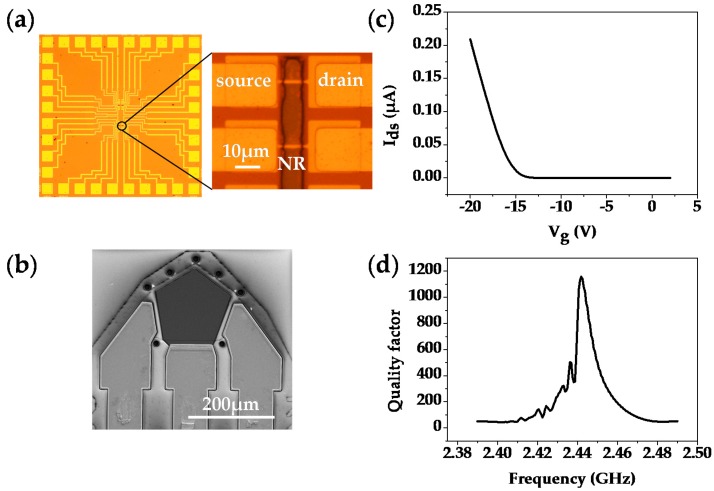
Illustration of the dual-mode gas sensor. (**a**,**b**) present the optical microscope images of the Si-NR FET array and the scanning electron microscopy (SEM) image of an FBAR configuration; (**c**) and (**d**) describe the I_ds_-V_g_ characteristic of the Si-NR FET sensor and the quality-factor curve of the FBAR sensor.

As mentioned above, the dual-mode gas sensor used in this work is composed of a Si-NR FET (Figure 3a) and a FBAR sensor (Figure 3b). Owing to the miniaturization and CMOS-compatible fabrication, both sensors show potential for large-scale integration into portable dual-mode sensing systems. Figure 3c presents the I_ds_-V_g_ curve of the Si-NR FET sensor. The device shows a typical p-type behavior, which means that the channel carrier density is tuned with a negative bias voltage applied to the back-gate. Thus, the adsorption of polar molecules can be directly detected in the accumulation mode of the device due to the surface potential change. In contrast, the sensing mechanism of the FBAR sensor is based on the mass loading effect, and the resonant frequency decreases with the loading of analytes. According to the Sauerbrey equation [44], high operation frequency gives the FBAR sensor a much higher sensitivity than conventional quartz crystal microbalance (QCM) [45]. The quality factor of a resonator is a parameter that describes energy losses over time, and is related to the sensing performance of the device [46]. Figure 3d presents the quality-factor curve of FBAR. The highest quality-factor is 1158 at the operation frequency of 2.44 GHz, indicating that the acoustic energy is well-trapped in the piezoelectric layer.

**Figure 4:**
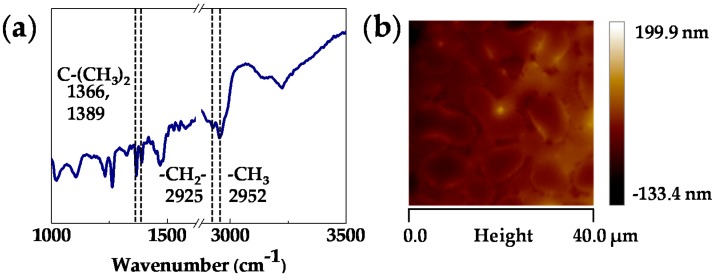
(**a**) FTIR spectroscopy result and (**b**) AFM image of the PIB layer coated on the FBAR sensor.

In order to further improve the sensitivity of the bare FBAR sensor [28], a polymer layer of PIB [47] was spin-coated on top of the device. The surface was characterized with FTIR spectroscopy and AFM. Figure 4 presents the characterization results of the PIB layer. The spectrum (Figure 4a) shows reflection peaks at 1366 and 1389 cm^−1^ due to symmetrical deformation vibrations of C-(CH_3_)_2_ as well as reflection peaks at 2925 and 2952 cm^−1^ due to the asymmetrical stretching vibrations of -CH_2_- and -CH_3_. In the AFM image (Figure 4b), a thickness of 58 nm and separate domains with 6~8 µm gaps can be found. These characterization results confirm a uniform coating of PIB on the FBAR sensor.

### 3.2. Si-NR FET Results

**Figure 5:**
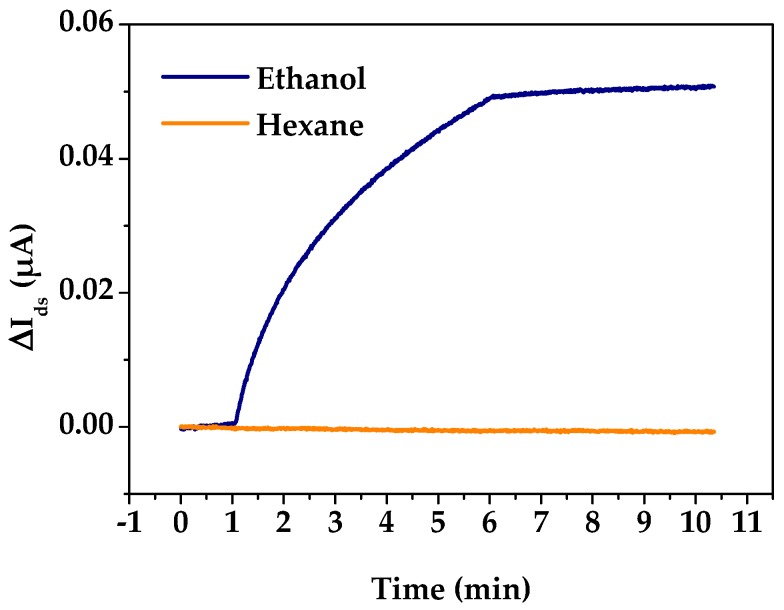
Real-time responses of a Si-NR FET device to ethanol and hexane at P/P_0_ = 0.1.

In the dual-mode gas sensor, the Si-NR FET sensor was incorporated to provide polarity information for the analytes. We first examined the influence of polarity on the FET response using ethanol and hexane as test samples. Figure 5 shows the real-time detection results of polar ethanol (dipole moment = 1.85) and nonpolar hexane (dipole moment = 0) at the same concentration with a Si-NR FET device. With the introduction of ethanol at 1 min, the drain current of the Si-NR FET sensor significantly increases. In contrast, the drain current does not change even with a continuous flow of hexane. The results confirm the polarity-sensitivity of the Si-NR FET sensor. For a Si-NR FET device, the response of the device depends on carrier concentration inside the Si-NR, which can be directly modulated by electrostatic interaction between a polar vapor molecule and the device. The long response time of the Si-NR FET sensor to ethanol is attributed to the chemical reaction kinetics between molecules and oxygen ions adsorbed on the surface of the nanomaterials [48]. In the following tests, the response time was shortened to 1 min, which still met the demand of detection.

**Figure 6:**
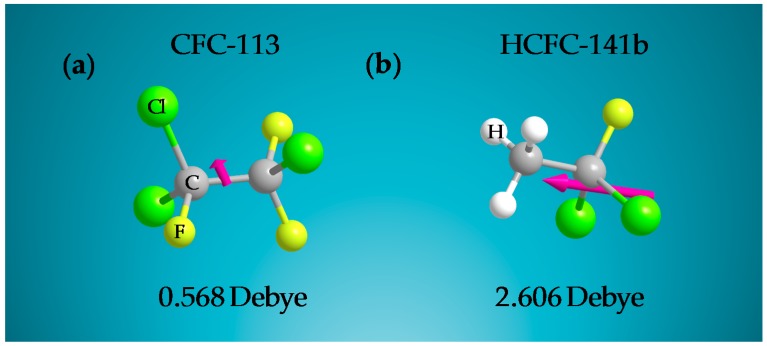
Three-dimensional (3D) molecular structure of (**a**) CFC-113 and (**b**) HCFC-141b. The magenta arrow indicates the orientation and value of the dipole moment for each structure based on calculations using ChemBio3D with the GAMESS interface.

We then simulated CFC-113 and HCFC-141b using ChemBio3D with the GAMESS interface [49]. In general, the properties of CFC-113 and HCFC-141b are found to be quite similar except for polarity. Figure 6 shows three-dimensional (3D) molecular structures of the two freons with their dipole moments. The simulation results indicate that HCFC-141b has higher polarity due to the partially halogenated structure, while CFC-113 has negligible polarity due to the nearly symmetric halogenated groups.

**Figure 7:**
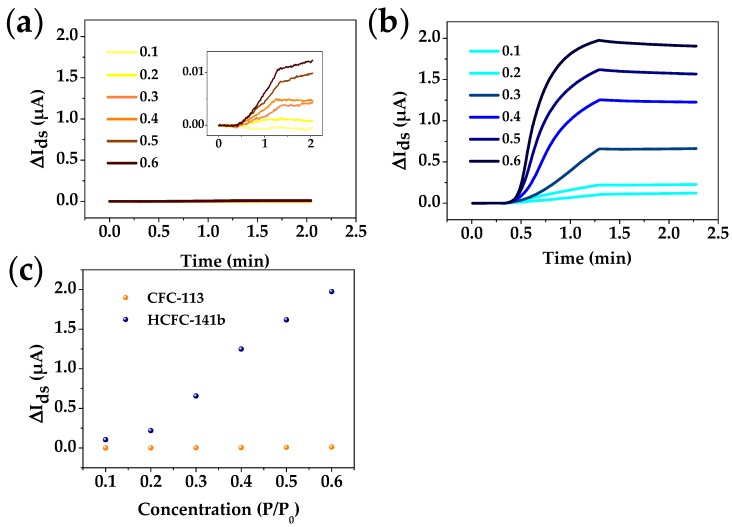
Plots of real-time responses of the Si-NR FET sensor to (**a**) CFC-113 and (**b**) HCFC-141b in the same scale at different concentrations (responses of the Si-NR FET sensor to CFC-113 in the appropriate scale are shown in the insert); (**c**) I_ds_ shifts of the Si-NR FET sensor at different concentration ranges.

Figure 7a,b show the real-time responses of the Si-NR FET sensor to CFC-113 and HCFC-141b vapors at different concentrations. The y-axis scale is kept the same between the two figures for comparison. It can be seen that the measured current (I_ds_) significantly increases once the sensor is exposed to HCFC-141b, while for CFC-113 the current keeps nearly constant during the entire sampling period. As discussed previously, the poor response of the Si-NR FET sensor to CFC-113 can be attributed to the weak polarity of the analyte. In contrast, a stronger polarity of HCFC-141b results in a much more obvious response from the Si-NR FET sensor (Figure 7c). These results suggest that CFC-113 and HCFC-141b can be effectively distinguished with the Si-NR FET sensor.

### 3.3. FBAR Results

**Figure 8:**
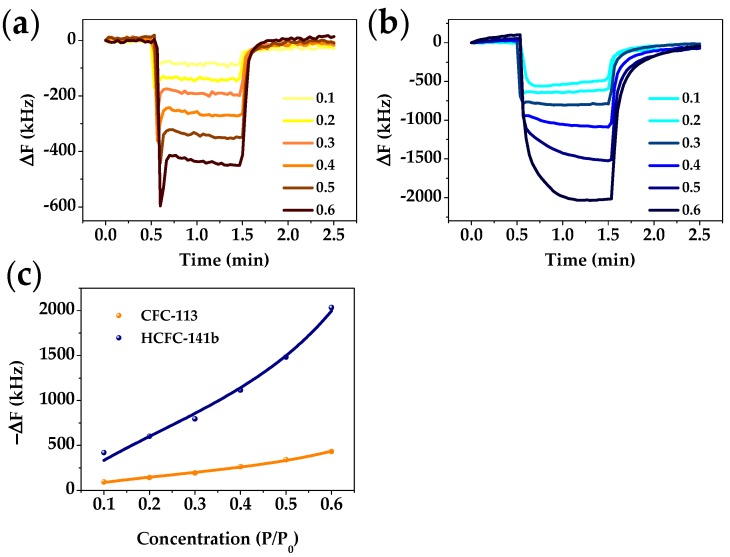
Plots of real-time responses of the FBAR sensor to (**a**) CFC-113 and (**b**) HCFC-141b at different concentrations from P/P_0_ = 0.1 to 0.6; (**c**) BET fitting of adsorption isotherms of freons on the PIB layer.

In order to obtain quantitative concentration information for freons regardless of polarity, we applied a PIB-coated FBAR for CFC-113 and HCFC-141b detection. Figure 8a,b respectively show the real-time responses of FBAR to CFC-113 and HCFC-141b vapor from P/P_0_ = 0.1 to 0.6. As can be seen from comparing the figures, HCFC-141b shows larger frequency shifts than CFC-113. To further understand this phenomena, we investigated the adsorption isotherms by fitting data with the Brunauer–Emmett–Teller (BET) equation [50], a typical model of multilayer gas physisorptions:

$$v=\frac{v_{m}\left(\frac{P}{P_{0}}\right)C}{1+\left(C-2\right)\left(\frac{P}{P_{0}}\right)-\left(C-1\right){\left(\frac{P}{P_{0}}\right)}^{2}}$$

where v, v_m_, P, and P_0_ are the adsorption capacity, the monolayer adsorption capacity, the vapor pressures, and the saturated vapor pressure of freons, respectively, and C is the adsorption energy constant. The excellent fit of the BET equation to the frequency data shown in in Figure 8c indicates that freons molecules are mainly adsorbed on the surface of PIB by multilayer molecule stacking at high concentration [51].

**Figure 9:**
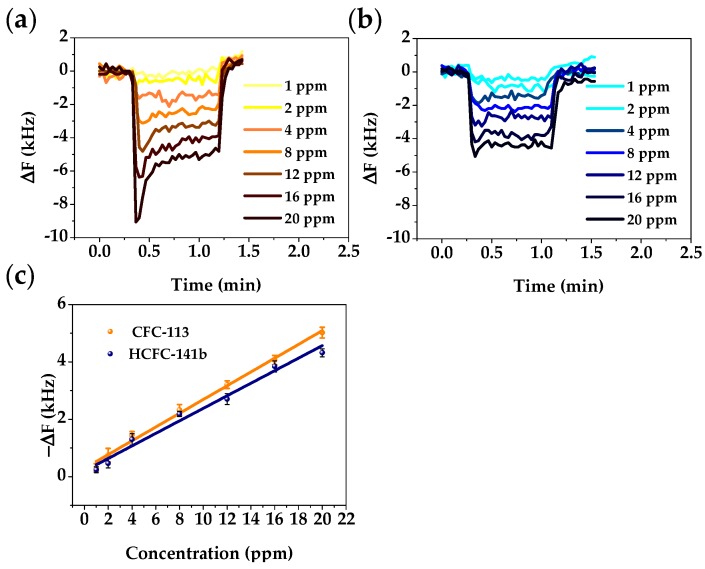
Plots of real-time responses of the FBAR sensor to (**a**) CFC-113 and (**b**) HCFC-141b at concentrations from 1 to 20 ppm; (**c**) Linear fits of responses of the FBAR sensor to freons at low concentration ranges.

To investigate the LOD of this method, the same sensor was used to detect a lower concentration range of CFC-113 and HCFC-141b (here we used the ppm horizontal axis instead of P/P_0_). Figure 9a,b respectively show the real-time responses of the FBAR sensor to CFC-113 and HCFC-141b vapors at 1 to 20 ppm. It is clear that the relationship between concentration and frequency change is linear within this concentration range (Figure 9c). This linearity is caused by the unsaturated adsorption of freons in the PIB polymer, and thus this adsorption can be modeled as monolayer adsorption. Owing to the high adsorption capacity of the polymer coating and high operation frequency of the device, the detection limit, typically calculated as three times the baseline noise level response, is estimated to be around 4 ppm.

It is worthwhile to point out that the frequency response to HCFC-141b at high concentration is larger than that of CFC-113 and this is opposite to the trend at low concentration. This is likely due to the fact that the freons form multilayer adsorptions on the sensor’s surface when exposed to high concentrations. In comparison, the adsorption of freons in the PIB polymer adopts a monolayer adsorption model at low concentration. In the monolayer adsorption case, the interaction of freons with PIB dominates, with both molecules showing similar behaviors, while for the multilayer stacking case, the interactions of freons with themselves dominate, with CFC-113 and HCFC-141b showing a large difference.

### 3.4. Mixture Analysis with the Dual-Mode Gas Sensor

**Figure 10:**
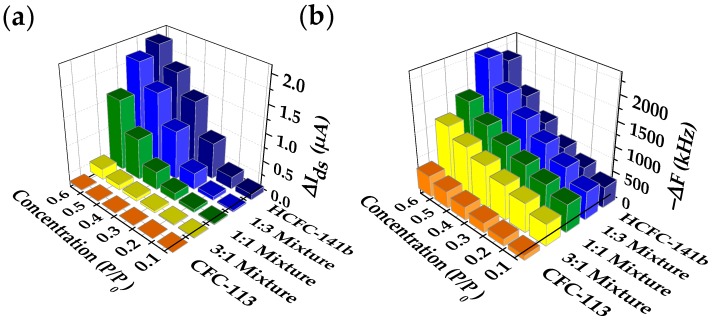
Freons mixtures detection by the dual-mode gas sensor. Three-dimensional bar plot of (**a**) current responses of the Si-NR FET sensor and (**b**) frequency responses of the FBAR sensor to CFC-113, HCFC-141b, and their three mixtures at different concentration ranges.

To further investigate the performance of the dual-mode gas sensor, here we demonstrate the qualitative and quantitative analysis of freons mixtures with the integrated complementary sensor array. Analytes in this study were prepared by bubbling nitrogen gas through liquid mixtures of CFC-113 and HCFC-141b with volume ratios of 3:1, 1:1, and 1:3. Figure 10 displays the results of CFC-113, HCFC-141b, and their mixtures measured by the dual-mode gas sensor. As shown in Figure 10a, the current response of the Si-NR FET sensor shows a strong positive correlation with the HCFC-141b proportion in the freons mixture, which indicates that the component of freon analyte can be directly discriminated with the device. Once the component is discriminated, concentration detection is achieved with FBAR-based mass detection. As shown in Figure 10b, BET correlations can be found for all vapors, confirming the excellent quantitative detection capacity of the FBAR sensor. The combination of complementary miniaturized sensors provides the dual-mode gas sensor with potential to become an integrated sensing system. Additionally, the novel gas sensor also holds potential for the analysis of other dual-component gas mixtures composed of polar and nonpolar vapor in the future.

## 4. Conclusions

In summary, a novel dual-mode gas sensor composed of a charge-sensitive Si-NR FET sensor and a mass-sensitive FBAR sensor was developed. Owing to the different sensing mechanisms, these two types of sensors offer complementary information regarding the analytes. Such system was successfully applied for the detection of CFC-113 and HCFC-141b. Because of the nonpolar property of CFC-113, it shows negligible current response on the Si-NR FET sensor, while the Si-NR FET sensor is very sensitive to the polar HCFC-141b. Thus, these two types of freon molecules can be effectively distinguished with the gas sensor. In addition, the polymer-coated FBAR sensor shows excellent quantitative detection capacity of the freon molecules with a ppm-level LOD. Mixtures of CFC-113 and HCFC-141b have been successfully analyzed with the dual-mode gas sensor. Once the component is discriminated by the Si-NR FET sensor, concentration information can be measured by the FBAR sensor. In conclusion, the sensing system can discriminate CFC-113 and HCFC-141b and calculate the concentration of each component by simple math without using a complex mathematic model. Therefore, it provides a much more effective method to distinguish the two components with different polarities than a sensing system based on one type of sensor, e.g., a conventional FBAR-based electronic nose. Though freon is used as a test compound to demonstrate the polarity differential, the sensing performance of the system can be further improved by arraying multiple Si-NR FETs as well as FBARs and coating the sensors with multiple chemicals (e.g., molecularly imprinted polymers [52]). Owing to the small size and CMOS-compatibility of the two types of sensors, the dual-mode sensing system is a potential candidate as a portable integrated analysis system for the analysis of gas mixtures.

## Figures and Tables

**Table 1:** Atmospheric lifetimes, ozone depletion potentials (ODPs), global warming potentials (GWPs) [40,41], and other typical physical properties of CFC-113 and HCFC-141b.

Freon	Atmospheric Lifetime (Years)	ODP	GWP	Volume ^1^ (Å^3^)	Boiling Point (K)	Critical Pressure (MPa)
CFC-113	93	0.81	6080	96.142	320.754	3.820
HCFC-141b	9.4	0.11	717	74.304	300.129	4.386

^1^ Volume was calculated as the Connolly solvent excluded volume.
